# Exchangeable Femoral Neck (Dual-Modular) THA Prostheses Have Poorer Survivorship Than Other Designs: A Nationwide Cohort of 324,108 Patients

**DOI:** 10.1007/s11999-017-5260-6

**Published:** 2017-02-13

**Authors:** Sandrine Colas, Assia Allalou, Antoine Poichotte, Philippe Piriou, Rosemary Dray-Spira, Mahmoud Zureik

**Affiliations:** 1 French National Agency for Medicines and Health Products Safety, Saint-Denis, France; 2 Centre Hospitalier Loire Vendée Océan, Challans, France; 3 Clinique du Parc, Autun, France

## Abstract

**Background:**

Exchangeable neck stems, defined as those with a dual taper (that is, a modular junction between the femoral head and the femoral neck and an additional junction between the neck and the stem body), were introduced in THA to improve restoration of joint biomechanics (restoring anteversion, offset, and limb length) and reduce the risk of dislocation. However exchangeable necks have been reported to result in adverse effects such as stem fractures and acute local tissue reaction. Whether they result in a net improvement to or impairment of reconstructive survivorship remains controversial.

**Questions/Purposes:**

(1) To compare the prosthetic survivorship and all-cause revision risk of exchangeable femoral neck THAs versus fixed neck THAs, taking known prosthetic revision risk factors into account; and (2) to compare the cause-specific revision risk of exchangeable femoral neck THAs versus fixed neck THAs, adjusting for known prosthetic risk factors.

**Methods:**

Using French national health-insurance databases, we identified all French patients older than 40 years who underwent primary THA from 2009 through 2012. To ensure accuracy of the data, we considered only beneficiaries of the general insurance scheme (approximately 77% of the population). Characteristics of the prosthesis and the patients receiving an exchangeable femoral neck THA were compared with those receiving a fixed femoral neck THA (defined as femoral stem with only the head being exchangeable). Revision was the event of interest. Followup started on the date the THA was performed, until the patient experienced revision, died, was lost to followup, or until the followup period ended (December 31, 2014), whichever came first. Competing risk THA survivorship was calculated and compared (purpose 1), as were cause-specific Cox regression models (purpose 2). The study cohort included 324,108 individuals with a mean age of 77 years. A total of 24% underwent THA for acute trauma, and 3% of the group received an exchangeable neck THA. During the median 45-month followup (mean, 42 months; minimum, 1 day; maximum, 6 years), 11,968 individuals underwent prosthetic revision.

**Results:**

The cumulative revision incidence was 6.5% (95% CI, 5.8%–7.3%) for exchangeable neck THAs versus 4.7% (95% CI, 4.6%–4.8%) for fixed neck THAs (p < 0.001). After controlling for potential confounding variables including age, sex, comorbidities, indication for THA, cementation, bearing surface, and the characteristics of the center where the implantation was performed, we found that the exchangeable femoral neck THA was associated with an increased hazard ratio (HR) of revision of 1.26 (95% CI, 1.14–1.38; p < 0.001) compared with the fixed neck THA. When dealing with cause-specific revision, exchangeable neck THAs had a higher incidence of revision for implant failure or periprosthetic fracture, and for mechanical complications; adjusted HRs were, respectively, 1.68 (95% CI, 1.24–2.27; p < 0.001) and 1.27 (95% CI, 1.13–1.43; p < 0.001), for exchangeable neck THAs compared with fixed ones.

**Conclusions:**

Exchangeable neck THAs had poorer survivorship independent of other prosthetic revision risk factors. Accordingly, expected anatomic and functional benefits should be carefully assessed before choosing this design.

**Level of Evidence:**

Level III, therapeutic study.

**Electronic supplementary material:**

The online version of this article (doi:10.1007/s11999-017-5260-6) contains supplementary material, which is available to authorized users.

## Introduction

Adjusting limb length, femoral offset, and implant positioning are all important to achieve a successful outcome of THA. Available techniques and technical options in the field of hip arthroplasty have been evolving for several decades; in the 1980s, exchangeability of the femoral neck was introduced to help surgeons in customizing the THA fit and matching the anatomic characteristics of the patient with better accuracy to improve ROM, stability, and abductor strength [23, 26, 42, 44]. Exchangeable neck stems—defined as those with modular junctions between the femoral head and the femoral neck and between the neck and the stem body—allow the surgeon to adjust limb length, femoral offset, and femoral anteversion independently from stem size or position.

Exchangeable necks are considered particularly useful to accommodate difficult cases of femoral deformity [35, 57], to restore joint biomechanics, and prevent prosthetic impingement-related complications. However, they also have been reported to result in adverse effects, including fretting, corrosion, implant failure, metallic wear debris generation [10, 19, 27, 30, 46], and local tissue reaction [10, 24, 29, 51]. Whether they result in a net improvement to or impairment of reconstructive survivorship remains controversial [8, 13, 14, 34, 50, 52]. Few studies have compared prosthetic survivorship of exchangeable neck versus fixed neck THAs, and findings are divergent [2, 14, 17]. The Australian Orthopaedic Association National Joint Replacement Registry reported exchangeable neck stem THAs have a higher rate of revision (almost twice) at 10 years compared with fixed neck stem THAs, in patients with osteoarthritis, regardless of the bearing surface [2]; Meftah et al. [33] also found a high cumulative revision rate with one specific model of exchangeable neck THA, but did not have a comparison group with fixed neck THA. Others found no difference [17, 18, 43, 50]. Except for the Australian registry from a population-based cohort (albeit one in which data on patients’ medical histories are limited), all other studies on this topic have been performed on small cohorts and often without a comparative group of fixed neck THAs. In addition, to our knowledge, none investigated prosthetic survivorship of exchangeable neck versus fixed neck THAs according to the implantation indication osteoarthritis (meaning degenerative or posttraumatic arthritis) or traumatic indication, and none explored causes for revision.

We therefore sought (1) to compare the prosthetic survivorship and all-cause revision risk of exchangeable femoral neck THAs versus fixed neck THAs, taking known prosthetic revision risk factors into account; and (2) to compare the cause-specific revision risk of exchangeable femoral neck THAs versus fixed neck THAs, adjusting for known prosthetic risk factors.

## Patients and Methods

We retrospectively used the French Health Insurance Information System (SNIIRAM), which has been validated [3, 20–22, 31, 37] and used in many studies [4–6, 9, 15, 28, 31, 32, 48, 49, 58, 59]. In France, health insurance is compulsory and it comprehensively covers the entire French population. It is divided into three main schemes: (1) general scheme covering employees in the industry, business, and service sectors, and some categories of workers considered as employees; (2) agricultural scheme covering farmers and farm employees; and (3) social scheme for independent professionals covering craftspeople, retailers, manufacturers, and independent workers. In our study, only the general scheme beneficiaries were included (approximately 77% of the population), because of technical reasons: for beneficiaries of other schemes, some information regarding medical details, long-term disease, or date of death do not follow the same recording process in the databases and are available only partially or with long delays. For beneficiaries of the general scheme, the SNIIRAM records with dates, outpatient drugs (Anatomical Therapeutic Classification codes), medical devices, services, and procedures reimbursed. The database does not specifically link indications for use of a particular device, service, or procedure to a reimbursement code, but contains patients’ demographic, administrative, and medical details (chronic conditions such as diabetes mellitus, cancer, or cardiovascular disease), and date of death. An anonymous, unique patient identifier links SNIIRAM information to national hospital discharge databases (Programme de Médicalisation des Systèmes d’Information [PMSI]), providing reasons for admission and discharge diagnoses (using International Statistical Classification of Diseases, 10th Revision [ICD-10]).

**Fig. 1:**
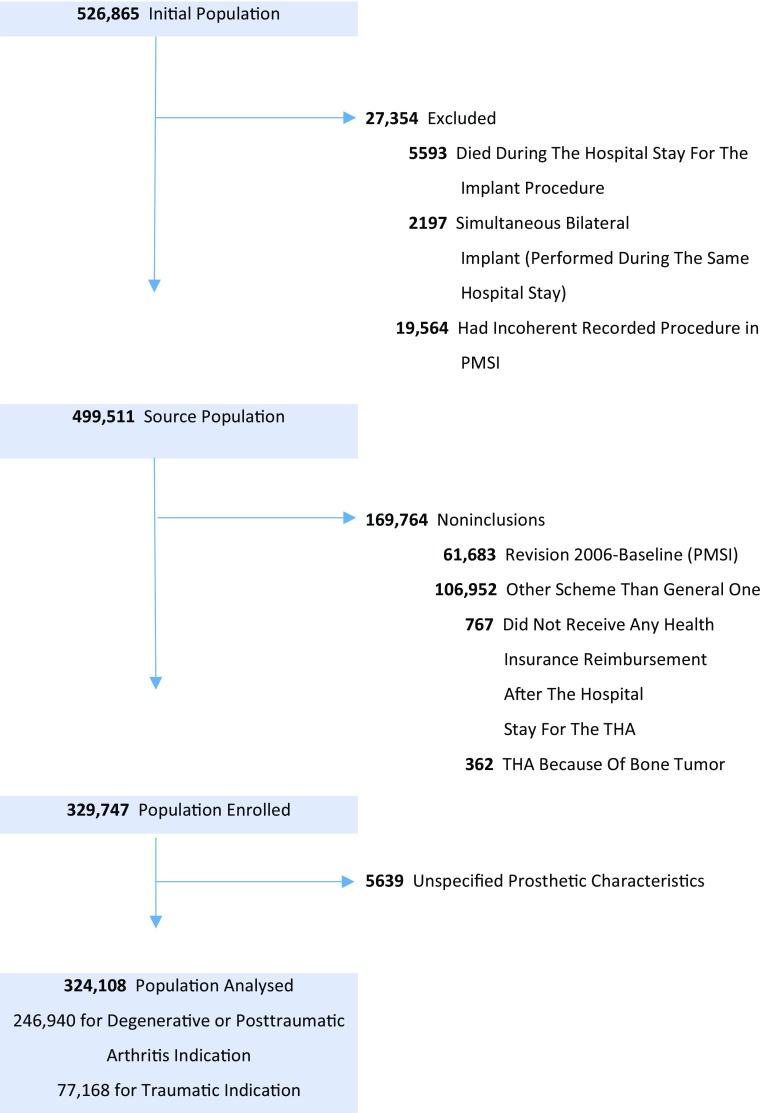
A flow chart of our study population is shown. PMSI = Programme de Médicalisation des Systèmes d’Information.

A population-based cohort of patients having primary THA was identified by the hospitals’ procedure claims and medical devices reimbursed; this method has been used and validated [20, 21]. The eligible population was all patients 40 years or older, having undergone unilateral primary THA between January 1, 2009, and December 31, 2012 (48 months). Patients having received primary THA for bone cancer, prosthetic revision before the index date, simultaneous bilateral THA, not having received any reimbursement 6 months after THA (therefore impossible to followup: n = 767; 0.2%), or with incoherent data in the PMSI were excluded (n = 19,564; 3.8%). THA characteristics were missing for 5639 (1.7%), who were excluded from subsequent analyses, leaving 324,108 (Fig. 1), among which 246,940 received implants for osteoarthritis and 77,168 received implants owing to acute trauma; 79,605 were enrolled in 2009, 80,226 in 2010, 81,654 in 2011, and 82,623 in 2012. Twenty five thousand four hundred seventy-three patients (7.9%) were lost to followup (their mean followup was 908 days, versus 1436 days for patients followed up to December 31, 2014).

Approval was obtained from the French data protection agency (Commission Nationale de l’Informatique et des Libertés). Informed consent was not required because information was collected anonymously.

**Fig. 2A–B:**
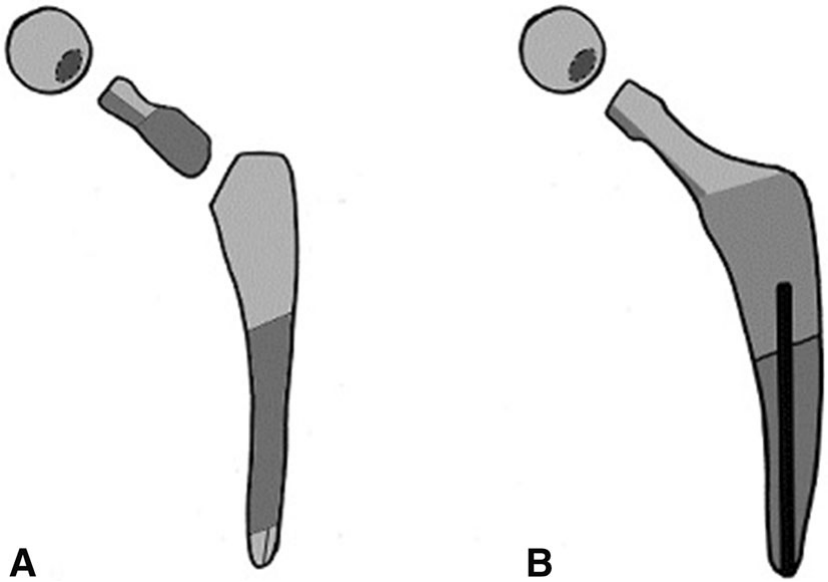
An **(A)** exchangeable femoral neck stem and **(B)** a fixed femoral neck stem are shown. An exchangeable femoral neck stem has a trunnion between the neck and ball head, and an additional trunnion between the neck and body of the stem. A fixed femoral neck stem has a trunnion only between the neck and ball head.

Two types of necks were considered: an exchangeable femoral neck and a fixed femoral neck. Exchangeable femoral necks were defined as a femoral stem with a dual taper, meaning a trunnion between the femoral head and neck, and an additional trunnion between the neck and body of the stem. Fixed femoral neck implants were defined as femoral stems with only the femoral head being exchangeable (that is, the stem body and neck are monolithic, but the head is modular) (Fig. 2).

The primary outcome was THA revision (including any surgical revision in which the implant or any component was changed or removed), regardless of the cause for that revision. We also identified causes for revision with algorithms based on the reason for admission, procedures coded in hospital claims, and medications reimbursed. We classified them as revision “for implant failure or periprosthetic fracture,” “for dislocation,” “for infection,” or “for mechanical complication” (including aseptic loosening, osteolysis, corrosion, adverse tissue reactions). When it was not possible to identify the cause for revision, we stated “unspecified cause.” Followup started at the date the THA was performed (index date) until the patient underwent revision surgery, was lost to followup (not receiving any medical care reimbursements recorded in the databases), died, or until the followup ended (December 31, 2014), whichever came first. Median followup was 45 months (mean, 42 months; minimum, 1 day; maximum, 6 years). Patient death was considered a competing risk.

We collected a series of patient, implantation center, and THA characteristics known to be or suspected of being associated with a risk of postarthroplasty complications. Information regarding patients’ age, sex, and date of death came from the SNIIRAM database. Treatments were identified by prescriptions (Anatomical Therapeutic Classification codes) reimbursed at least once within 180 days before or after inclusion, namely antidepressants, antihypertensives, oral corticosteroids, osteoporosis treatments, psychostimulants, antiepileptics, benzodiazepines, anxiolytic or hypnotic nonbenzodiazepines (non-BZD), and antipsychotics. Diabetes mellitus, morbid obesity (corresponding to a BMI greater than 30 kg/m^2^), Parkinson’s disease, immunodeficiency, and chronic kidney disease were defined (ICD-10 categories) on the basis of hospital discharge reports or chronic condition recorded the year before inclusion, with relevant prescriptions. The indication for THA (osteoarthritis or traumatic indication) was identified based on hospital discharge reports. The mean number of THAs performed per month (during the 4-year inclusion period) was calculated. Whether centers where the THAs were performed were private or public and the duration of hospital stay (in days) also were collected. Four types of THA fixation techniques (uncemented, both sides cemented, hybrid [femoral component cemented, acetabular component uncemented], and reverse hybrid [femoral component uncemented, acetabular component cemented]), and four different bearings (ceramic-on-ceramic [CoC], ceramic-on-polyethylene [CoP], metal-on-metal [MoM], and metal-on-polyethylene [MoP]) were analyzed.

### Statistical Analysis

Cumulative incidence of revision (whatever the cause) was represented according to type of femoral neck using a Fine and Gray [16] proportional hazards regression model with death as a competing risk. Hazard ratios (HRs) for revision according to the type of femoral neck were assessed using univariate and multivariate Fine and Gray proportional hazards regression models adjusting for possible confounding factors: sex; age category at implantation: young (40–59 years), middle-aged (60–74 years), or elderly (≥ 75 years); indication for implantation (osteoarthritis, traumatic); diabetes mellitus; morbid obesity; Parkinson’s disease; immunodeficiency; medication (antidepressants, oral corticosteroids, antiosteoporotics, psychostimulants, benzodiazepines, non-BZD antiepileptics, non-BZD anxiolytic/hypnotic, antipsychotics); public or private sector; center activity volume (tertiles); hospital stay duration (three groups: < 6 days; 6–12 days; > 12 days); cement type (four categories); and bearing surface (four categories). These characteristics were included simultaneously in the multivariate Fine and Gray proportional hazards regression model [16].

We also fitted cause-specific Cox proportional hazards regression models for the following five indications for revision: implant failure or periprosthetic fracture, dislocation, infection, mechanical complication, and other cause, and we estimated cause-specific adjusted HRs. Assumption of proportional hazards was graphically assessed for each variable. Interactions between exposure and age and sex, indication, cement type, and bearing surface in association with prosthetic survivorship were investigated, and we performed analyses stratified on sex, age group, indication, cementation type, and bearing surface.

### Cohort Description at Inclusion

The median age of the 324,108 patients included was 74 years (interquartile range, 64–81 years; mean, 72.6 years; SD, 11.7 years). Twenty-four percent of patients underwent THA for a traumatic indication. Sixty-two percent of the enrolled patients were women, and more likely received THA for a traumatic indication (29% versus 15% for men, p < 0.001), and were older than the men (75 versus 69 years, p < 0.001) (Supplemental Table 1. Supplemental materials are available with the online version of *CORR* ^*®*.^). Implantation was performed at a private-sector hospital in 58% of patients and 71% of procedures were performed in centers in which more than 14 procedures per month were done. Fixation was uncemented in 71% of patients, cemented in 11%, hybrid in 17%, and reverse hybrid in 2%. Bearing surfaces were CoC (32%), CoP (17%), MoM (3%), and MoP (48%). We also reported characteristics at inclusion, according to sex and indication for THA (Supplemental Table 1. Supplemental materials are available with the online version of *CORR* ^*®*.^).

An exchangeable femoral neck was implanted in a total of 8931 (3%) patients, with a stable proportion with time: 2.7% of patients included in 2009, 2.9% in 2010, 2.7% in 2011, and 2.8% in 2012. We reported patient characteristics, hospital stay, and bearing surface at inclusion, according to the type of femoral neck (Table 1). Exchangeable neck THAs are performed more frequently in young patients and in patients not experiencing trauma, and are performed mostly in public hospitals. Implants with neck exchangeability also are associated with other THA characteristics: they are used more frequently with MoM and CoC bearing surfaces and with uncemented THAs. Type of femoral neck was strongly associated with the hospital where the THA was performed. Among 891 centers where implants were performed, more than 5% of exchangeable neck THAs were done in 100 centers (globally, 21% of exchangeable neck THAs were performed at these 100 centers versus 0.47% among the 791 others; p < 0.0001). These 100 centers were more likely to be public hospitals (nine were teaching hospitals) with more than 38 procedures being performed per month. The characteristics of patients receiving THAs at centers performing low numbers of exchangeable neck implants versus at centers performing high numbers of exchangeable neck implants were similar.

**Table 1:** Baseline characteristics according to type of femoral neck

		Fixed neck (%)	Exchangeable neck (%)	p Value*
Covariates	Number	(n = 315,177)	(n = 8931)	
THA characteristics				
Cement type^†^				< 0.001
Cemented	34,376	11	3	
Hybrid	53,611	17	9	
Reverse hybrid	5040	2	2	
Uncemented	231,081	71	86	
Bearing surface				< 0.001
CoC	104,584	32	46	
CoP	56,055	17	14	
MoM	8667	3	6	
MoP	154,802	48	35	
Patient characteristics				
Sex				< 0.001
Male	122,178	38	42	
Female	201,930	62	58	
Age category (years)				< 0.001
40–59	46,945	14	21	
60–74	122,590	38	43	
≥ 75	154,573	48	37	
Trauma indication	77,168	24	16	< 0.001
Parkinson disease	13,158	4	3	< 0.001
Diabetes mellitus	40,475	13	13	0.18
Morbid obesity	24,678	8	8	0.03
Treatments				
Benzodiazepine	163,289	50	50	0.24
AH no BZD	45,534	14	14	0.37
Antidepressant	73,418	23	21	< 0.001
Antipsychotic	22,364	7	6	< 0.001
Psychostimulant	3386	1	1	0.62
Antiosteoporotic	42,311	13	12	0.02
Oral corticosteroïds	83,198	26	27	0.02
Hospital characteristics				
Sector				< 0.001
Public	136,853	41	71	
Private	187,255	59	29	
Number of THAs per month				< 0.001
< 14	93,647	49	42	
14–38	158,262	29	33	
> 38	72,199	22	25	
Hospital stay duration (days)				< 0.001
< 6	15,952	78	82	
6–12	254,695	5	5	
> 12	53,461	17	13	

*Exchangeable versus fixed femoral neck THAs; ^†^percentages for fixed neck cement types = 101% owing to rounding; CoC = ceramic-on-ceramic; CoP = ceramic-on-polyethylene; MoM = metal-on-metal; MoP = metal-on-polyethylene; AH no BZD = anxiolytic or hypnotic nonbenzodiazepines.

## Results

### Survivorship and All-cause Revision

**Fig. 3:**
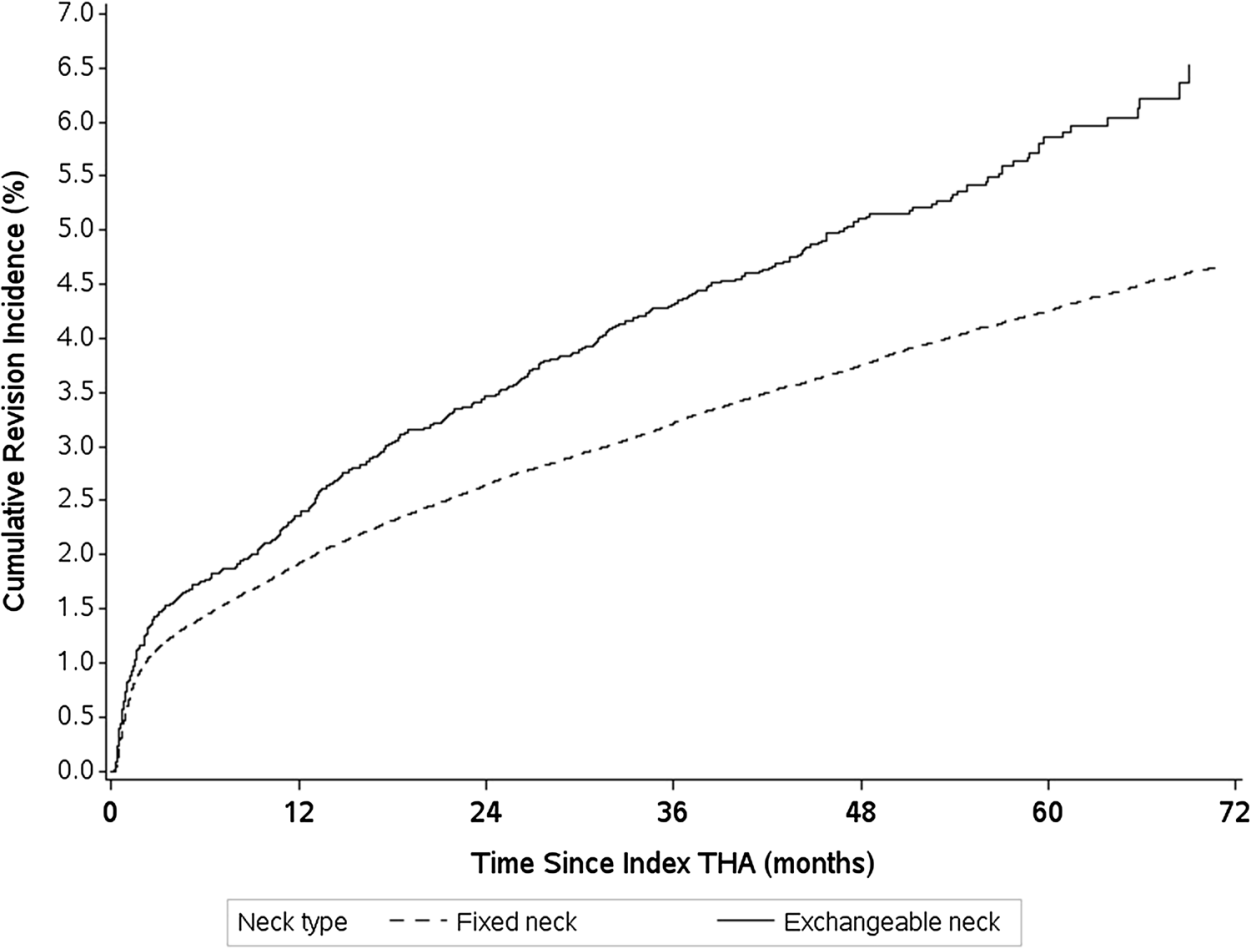
The cumulative incidence of revision THA according to the type of femoral neck is shown.

Patients receiving exchangeable neck stem implants were more likely to undergo revision than those with fixed neck stems designs (HR, 1.36; 95% CI, 1.24–1.49; p < 0.001). The cumulative incidence of prosthetic revision was 7% for THAs with exchangeable neck implants versus 5% for THAs with fixed neck implants (Fig. 3). After controlling for potential confounding variables such as patient age, sex, comorbidities, indication for THA, cementation, bearing used, and the center characteristics, we found that implantation of an exchangeable femoral neck design was associated with an increased adjusted HR of revision of 1.26 (95% CI, 1.14–1.38; p < 0.001) compared with fixed neck design (Table 2). This association had the same pattern for both implantation indications: adjusted HRs for revision for exchangeable neck THAs were 1.25 (95% CI, 1.13–1.39) and 1.19 (95% CI, 0.94–1.51) compared with fixed neck THAs in patients implanted for an indication of osteoarthritis or a traumatic indication, respectively. Other characteristics, including gender, age, indication for implantation, medications apart from psychostimulant and antiosteoporotic drugs, and center activity, were associated with prosthetic survivorship after controlling for all studied covariates; revision risk also was greater in patients who received an implant for a traumatic indication (Table 2).

**Table 2:** Associations among THA, patients, hospital stay characteristics, and THA revision

Covariates	Values	Number	Revision (%)	HR	95% CI	p Value	Adjusted HR*^,†^	95% CI	p Value
THA characteristics									
Exchangeable neck	No	315,177	3.7	1	Reference	< 0.0001	1	Reference	< 0.001
	Yes	8931	4.9	1.36	(1.24–1.49)		1.26	(1.14–1.38)	
THA cement type	Cemented	34,376	3.0	1	Reference	< 0.0001	1	Reference	< 0.001
	Hybrid	53,611	2.9	0.94	(0.87–1.02)		0.99	(0.92–1.08)	
	Reverse hybrid	5040	4.5	1.52	(1.32–1.76)		1.53	(1.33–1.77)	
	Uncemented	231,081	4.0	1.31	(1.23–1.40)		1.31	(1.22–1.40)	
Bearing surface	CoC	104,584	4.0	1.12	(1.07–1.16)	< 0.0001	1.07	(1.01–1.13)	< 0.001
	CoP	56,055	3.5	0.99	(0.94–1.04)		1.02	(0.96–1.08)	
	MoM	8667	5.0	1.30	(1.18–1.44)		1.25	(1.12–1.38)	
	MoP	154,802	3.5	1	Reference		1	Reference	
Patient characteristics									
Sex	Male	122,178	3.8	1.05	(1.02–1.09)	0.004	1.09	(1.05–1.14)	< 0.001
	Female	201,930	3.6	1	Reference		1	Reference	
Age category (years)	40–59	46,945	4.7	1.24	(1.17–1.30)	< 0.0001	1.19	(1.13–1.25)	< 0.001
	60–74	122,590	3.8	1	Reference		1	Reference	
	≥ 75	154,573	3.3	0.89	(0.85–0.92)		0.86	(0.82–0.90)	
Trauma indication	No	246,940	3.7	1	Reference	< 0.0001	1	Reference	< 0.001
	Yes	77,168	3.8	1.09	(1.05–1.14)		1.12	(1.06–1.19)	
Parkinson disease	No	310,950	3.6	1	Reference	< 0.0001	1	Reference	< 0.001
	Yes	13,158	4.9	1.39	(1.28–1.50)		1.26	(1.16–1.37)	
Diabetes mellitus	No	283,633	3.7	1	Reference	0.24	1	Reference	0.491
	Yes	40,475	3.8	1.03	(0.98–1.09)		1.02	(0.97–1.08)	
Morbid Obesity	No	299,430	3.6	1	Reference	0.0004	1	Reference	< 0.001
	Yes	24,678	4.2	1.16	(1.09–1.24)		1.14	(1.07–1.22)	
Treatments									
BZD	No	160,819	3.0	1	Reference	< 0.0001			< 0.001
	Yes	163,289	4.4	1.42	(1.37–1.47)		1.28	(1.23–1.33)	
AH no BZD	No	278,574	3.5	1	Reference	< 0.0001	1	Reference	< 0.001
	Yes	45,534	4.9	1.43	(1.36–1.49)		1.24	(1.18–1.30)	
Antidepressant	No	250,690	3.4	1	Reference	< 0.0001	1	Reference	< 0.001
	Yes	73,418	4.9	1.47	(1.41–1.53)		1.31	(1.25–1.37)	
Antipsychotic	No	301,744	3.6	1	Reference	< 0.0001	1	Reference	0.348
	Yes	22,364	4.6	1.30	(1.22–1.39)		1.03	(0.97–1.11)	
Psychostimulant	No	320,722	3.7	1	Reference	0.31	1	Reference	0.769
	Yes	3386	4.2	1.09	(0.92–1.29)		1.03	(0.87–1.21)	
Antiosteoporotic	No	281,797	3.7	1	Reference	0.02	1	Reference	0.008
	Yes	42,311	3.9	1.06	(1.01–1.12)		1.08	(1.02–1.14)	
Oral corticcosteroïds	No	240,910	3.5	1	Reference	< 0.0001	1	Reference	< 0.001
	Yes	83,198	4.2	1.20	(1.15–1.25)		1.13	(1.09–1.18)	
Hospital stay characteristics									
Sector	Public	136,853	3.7	1.05	(1.01–1.08)	0.02	1.02	(0.98–1.06)	0.453
	Private	187,255	3.7	1	Reference		1	Reference	
Number of procedures per month	< 14	93,647	3.7	1.20	(1.14–1.27)	< 0.0001	1.18	(1.12–1.24)	< 0.001
	14–38	158,262	4.0	1.09	(1.04–1.15)		1.09	(1.04–1.14)	
	> 38	72,199	3.3	1	Reference		1	Reference	
Hospital. stay duration (days)	< 6	15,952	3.7	0.99	(0.91–1.08)	0.001	1.00	(0.92–1.09)	0.198
	6–12	254,695	3.4	1	Reference		1	Reference	
	> 12	53,461	4.0	1.09	(1.04–1.15)		1.05	(1.00–1.10)	

*p value class versus reference; ^†^adjusted hazard ratio of THA revision from multivariate Fine and Gray full regression model (adjusted for THA characteristics, patient characteristics, treatments, and hospital stay characteristics); HR = hazard ratio; CoC = ceramic-on-ceramic; CoP = ceramic-on-polyethylene; MoM = metal-on-metal; MoP = metal-on-polyethylene; BZD = benzodiazepine; AH no BZD = anxiolytic or hypnotic nonbenzodiazepines.

During the median 45-month followup (mean, 42 months; minimum, 1 day; maximum, 6 years), 11,968 individuals underwent prosthetic revision. The prosthetic revision rate was 4%. The overall cumulative incidence for revision, when taking into account death as a competing risk, was 1.9% at 1 year, 2.7% at 2 years, 3.2% at 3 years, 3.8% at 4 years, 4.3% at 5 years, and 4.7% at 6 years followup. The median time-to-event for all-cause revision was 338 days (interquartile range, 56–816 days). Fifty-nine percent of revised exchangeable neck THA implants were replaced with fixed neck femoral stems (compared with 3% of primary fixed necks replaced with an exchangeable stem when revised). Among patients who had revision surgery, we also compared stem-specific revision (defined as revision of the head only, or head and neck, or head, neck, and stem) rate according to the type of neck; this rate was higher in exchangeable neck THAs compared with fixed neck THAs (39% versus 34%; p = 0.016).

### Cause-Specific Revision Risk

Patients who had THAs with an exchangeable neck implant were more likely to undergo revision for implant failure or periprosthetic fracture, and for mechanical complications (Table 3). Adjusted HRs for revision resulting from implant failure or periprosthetic fracture and adjusted HRs for revision resulting from mechanical complication were, respectively, 1.68 (95% CI, 1.24–2.27; p < 0.001) and 1.27 (95% CI, 1.13–1.43; p < 0.001) for exchangeable neck THAs compared with fixed ones. Similar results were observed when stratifying for gender, age group, indication, cementation type, and bearing surface (Supplemental Table 2. Supplemental materials are available with the online version of *CORR* ^*®*^.). Median time-to-event was, respectively, 93 (interquartile range [IQR], 32–498 days), 146 (IQR, 31–759 days), 210 (IQR, 30–708 days), 440 (IQR, 126–912 days), and 436 days (IQR, 170–850 days) for revisions resulting from dislocation, implant failure or periprosthetic fracture, infection, mechanical complication, and unspecified cause, respectively.

**Table 3:** Comparison of overall and cause-specific risks of THA revision according to type of THA femoral neck

Cause of revision	Frequency of THA revision				p Value	Cause-specific		p Value
	Patients with fixed neck (n = 315,177 [97%])		Patients with exchangeable neck (n = 8931 [3%])			Risk of revision associated with type of femoral neck		
	Number	Percent	Number	Percent		Adjusted HR^*^	95% HR CI	
All-cause	11,968	3.7	442	4.9	< 0.001	1.26	1.14–1.38	< 0.001
Periprosthetic fracture or implant failure	1050	0.3	45	0.5	< 0.001	1.68	1.24–2.27	< 0.001
Dislocation	2644	0.8	86	1.0	0.117	1.15	0.92–1.42	0.222
Infection	1345	0.4	37	0.4	0.992	0.95	0.69–1.33	0.954
Mechanical complication	7817	2.4	300	3.4	< 0.001	1.27	1.13–1.43	< 0.001
Other	285	0.1	15	0.1	0.052	1.62	0.96–2.76	0.073

Sum of different causes of revision (n = 13,141) > number of all-cause revisions (n = 11,968) because each revision could have multiple causes; ^*^cause-specific adjusted hazard ratio of THA revision from multivariate Cox full model (adjusted for THA characteristics, patient characteristics, treatments, and hospital stay characteristics); HR = hazard ratio.

## Discussion

Exchangeable neck stems have been used in THA to improve restoration of anteversion, offset, and limb length, and to reduce the risk of dislocation. However neck exchangeability has been reported to result in adverse effects such as stem fractures and acute local tissue reaction. Whether they result in a net improvement to or impairment of reconstructive survivorship remains controversial, with inconsistent results in relatively few comparative studies on the topic [2, 14, 17] (Table 4). In our study, which included a large, relatively unselected population, we found that the risk of revision was higher for exchangeable neck THAs compared with fixed ones. After controlling for potential confounding variables such as patient age, sex, comorbidities, indication for THA, THA bearing, cementation, and the center characteristics, we found that implantation of an exchangeable femoral neck THA was associated with an increased hazard ratio of revision compared with fixed neck THAs. In terms of cause-specific revision, exchangeable neck THAs had a higher incidence of revision for implant failure or periprosthetic fracture and of revision for mechanical complications.

**Table 4:** Registry and literature data for exchangeable and fixed femoral neck THA revision rates and risk

Source	Data period	Implant type	Cumulative revision rate (%)[95% CI]	Average followup (years)	Hazard ratio exchangeable vs fixed implant [95% CI]
	Number of THAs				
Australian Orthopaedic Association National Joint Replacement Registry [2]	2006–2014 Total = 988,667 (11.4% were revisions)	All THA	1.6 [1.6–1.7]	1	
			3.9 [3.9–4.0]	5	
			5.2 [5.1–5.3]	7	
			6.8 [6.7–6.9]	10	
		Exchangeable neck (all models)	8.3 [7.5–9.2]	7	2.04 [1.87–2.22]
		Fixed neck (all models)	3.7 [3.6–3.8]	7	1.00 [reference]
Swedish Hip Register, [54]	1979–2014 Total = 396,197 (13% revisions)	All THA*	6.2 [5.9–6.5]	10	NP
New Zealand Joint Registry [41]	1999–2012 Total = 98,500 (12.9% revisions)	All THA*	1.3 [NP]	1	NP
			3.3 [NP]	5	
			4.5 [NP]	7	
			6.9 [NP]	10	
National Joint Registry for England, Wales and Northern Ireland [38]		All THA*	0.8 [0.7–0.8]	1	
			2.7 [2.7–2.8]	5	
			4.0 [3.9–4.1]	7	
			5.8 [5.6–5.9]	10	
The Norwegian Arthroplasty Registry, [56]	1987–2014 Total = 190,962 (14.3% revisions)	All THA*	6.9 [6.6–7.2]	10	NP
Canadian Joint Replacement Registry [7]	2008–2013 Total = 216,358 (8.7% revisions)	All THA*	NP	NP	NP
Netherlands Arthroplasty Register [40]	2007–2012 Total = 114,110 (11.8% revisions)	All THA*	NP	NP	NP
Mihalko et al. [34]	Total = NP Review	Exchangeable neck^#^ (all models)	0 to 9% [NP]	10 to 19	NP
		Exchangeable neck^#^ (S-ROM)	16% [NP]	17	NP
Meftah et al. [33]	Total = 123 By one surgeon	Exchangeable neck^#^ (Rejuvenate)	28% [NP]	3	NP
Silverton et al. [52]	Total = 152 Single-center cohort	Exchangeable neck^#^ (Profemur Z)	10.6% [NP]	4.5	NP
Regis et al. [50]	Total = 168 66 Profemur R 102 Wagner SL	Exchangeable neck (Profemur R)	9.1%^§^	2	NP
		Fixed neck (Wagner SL)	6.8%^§^ (p = 0.4)		
Gerhardt et al. [18]	Total = 190 95 Profemur Z 95 Alloclassic Zweymüller	Exchangeable neck (Profemur R)	4%^§^	1	NP
		Fixed neck (Alloclassic Zweymüller)	4%^§^ (p = 0.4)		
Fitch et al. [17]	Total = NP 692 Profemur Z	Exchangeable neck (Profemur Z)	4.2% [2.6-5.8]	12	NP
		Fixed neck (All models)	3.9% [NP] (p = 0.36)		
Ollivier et al. [43]	Total = NP 170 Profemur Z	Exchangeable neck^#^ (Profemur Z)	NP	6	NP
Duwelius et al. [14]	Total = 878 594 Taper Kinectiv 284 Taper By one surgeon	Exchangeable neck (M/L Taper Kinectiv)	1% [0–2]	2	NP
		Fixed neck (M/L Taper)	1% [0–3]		
			(p = 0.94)		

*Cumulative revision rates for exchangeable neck only, and for fixed neck only not provided, which makes comparison impossible;^#^cumulative revision rate for fixed femoral neck not provided, which makes comparison impossible; ^§^cumulative risk for dislocation as outcome; NP = not provided

This study had several limitations. Regarding the implants, the alloys of the components (stem, exchangeable neck, and head) were not available, which would be interesting since the revision rate was found to be higher with a titanium alloy-cobalt alloy configuration [2]. In addition, the detailed design of the implant (such as the head diameter, surface finish, taper geometry, among others) was not available, nor was the brand of the implant; some models and designs of exchangeable neck implants seem to be better than others [2]. Consequently, our results might hide heterogeneity in the revision rates between the different kinds of exchangeable neck implants available on the market. Some specific exchangeable neck stem designs may offer similar survivorship to fixed neck stem designs. Nonetheless, we were interested in assessing a possible class issue regarding exchangeable neck stems, whatever the model. Our results showed exchangeable neck THAs have poorer survivorship than fixed neck ones, consistent with the results of the Australian registry [2], which found the same for all six exchangeable neck implants. Making the brand name of the THA implant available in hospital claims in the future might be of great interest. Other limitations were the lack of information regarding the surgical approach and use of dual-mobility bearing surfaces. This information was not available in the databases. We acknowledge that some complications, such as dislocations and periprosthetic fractures, are associated with the surgical technique [1, 11, 25], and dual-mobility articulations designed to reduce the risk of dislocation appear to be helpful [12], despite some remaining concerns about intraprosthetic dislocation [47].

Although we did not study stem-specific revision as the main outcome, we found in additional analysis that the stem-specific revision rate was higher for exchangeable neck THAs compared with fixed neck ones; the mechanism associated with these findings need to be confirmed in other studies. Among the hips revised for implant failure or periprosthetic fracture, we were not able to distinguish between periprosthetic fractures and implant failures. Although fractures of the neck are not uncommon with exchangeable femoral components, these events are probably mainly periprosthetic fractures, which represent one of the top five most-frequent causes for revision [61]. Regarding the mechanical complication designation, this covers a wide range of different types of failures and because of the nature of the data we used, we were not able to identify the mechanism having led to the revision. Previous studies were conducted to understand the mechanisms of failure related to the exchangeable neck implant. Corrosion at the exchangeable neck-body stem, fretting, or mechanically assisted crevice corrosion was identified as specifically associated with the femoral exchangeable neck, possibly resulting in adverse local tissue reactions [24, 30, 36, 39, 45, 51, 52]. Other typical findings include iliopsoas and abductor tendinopathy, peritendinous collections, and metallic debris, which might generate osteolysis [13, 29]. We assume some of the revisions resulting from mechanical complications in our cohort probably included these typical issues, although we were not able to identify them precisely. Finally, the accuracy of primary THA and revision procedure codes we used as inclusion and outcome criteria might be open to criticism. However, in our algorithm, we checked the agreement between the coded procedure and the implanted device to track for coding errors and we excluded the few patients (5.5%) with incoherent data. Moreover, this database is used to calculate payments for inpatient care with internal and external quality control processes. Coding errors, if any, would be marginal and likely would not differ among the study groups.

The overall risk of prosthetic revision observed after 45 months median followup is consistent with data from international registries, and supports the external validity of our study (Table 4). Likewise, the higher failure rate we found for exchangeable femoral neck THAs (Table 2) is consistent with rates in some previous studies (Table 4). Nonetheless, in the Australian registry, the risk of revision for exchangeable neck THAs was almost twice that of fixed neck THAs [2], an effect size much higher than what we observed. We speculate but cannot prove that this may be a function of the models, types, and brands of exchangeable neck implants used. The risk of revision varies from 3% to 18% at 5 years in the Australian registry [2], and was reported as much as 28% for one specific model [33]. We believe the lower (5%) revision rate we found might be the result of two poorly performing stems being rarely used in our population, and the exchangeable neck models with the highest failure rates in the Australian registry were not distributed in France. Revision risk also varies according to stem-neck interface material [2], with a titanium-cobalt chromium interface experiencing 1.5- to twofold higher risks of revision than a titanium-titanium interface. In addition to the Australian registry, two studies comparing THA survivorship according to type of neck both focused on only one model of exchangeable neck THA [14, 17] and included small cohorts, with findings opposite those of the Australian registry (Table 4). Our study therefore fills a gap in knowledge, not only because it is a nationwide “real-life” cohort, with different devices implanted, but also because we were able to control for important confounding factors in our analyses.

Regarding cause-specific revision, Wright et al. [60], Sporer et al. [53], and Talmo et al. [55] presented case reports of exchangeable femoral neck breakage or spontaneous dissociation. Our results regarding cause-specific revision risk extend this finding of higher risk of revision because of implant failure or periprosthetic fracture and because of mechanical complications in a large nationwide cohort. We found no association between revision attributable to dislocation and exchangeable neck THAs. Neck exchangeability was not found to be efficient in reducing the dislocation rate [50], yet restoration of offset and reducing the risk of dislocation are the main purposes of exchangeable femoral necks. Our work provides an answer regarding whether an exchangeable femoral neck results in a net improvement to or an impairment of reconstructive survivorship.

Exchangeable-neck THAs have a poorer survivorship independent of other prosthetic revision risk factors. If causal, it implies patients receiving exchangeable neck THAs are not given the best possible chances compared with patients receiving fixed neck THAs. Whatever the mechanism, expected anatomic and functional benefits should be assessed carefully before choosing this design, which might be reserved for patients with severe proximal femoral deformities that preclude the use of fixed neck femoral stems.

## Electronic supplementary material

Below is the link to the electronic supplementary material.

Supplementary material 1 (DOC 89 kb)

Supplementary material 2 (DOC 78 kb)
