# Supplementary material for: Exchangeable Femoral Neck (Dual-Modular) THA Prostheses Have Poorer Survivorship Than Other Designs: A Nationwide Cohort of 324,108 Patients
Source: Clin Orthop Relat Res. 2017 Feb 13;475(8):2046–59. doi: 10.1007/s11999-017-5260-6 (PMC5498370; doi:10.1007/s11999-017-5260-6)
Supplement: Supplementary file 1 — Supplementary material 1 (DOC 89 kb) [file 11999_2017_5260_MOESM1_ESM.doc]

**Supplemental Table 1.** Baseline characteristics according to gender and implantation indication

| Covariates | Values |  |  | Men | Women | Osteoarthritis | Traumatic |
| --- | --- | --- | --- | --- | --- | --- | --- |
|  |  | Percent | Number | (%) | (%) | (%) | (%) |
| THA characteristics |  |  |  |  |  |  |  |
| THA cement type* | Cemented | 11 | 34,376 | 7.1 | 12.7 | 5.8 | 26.0 |
|  | Hybrid | 17 | 53,611 | 14.7 | 17.6 | 19.3 | 7.7 |
|  | Reverse hybrid | 2 | 5040 | 1.1 | 1.8 | 1.5 | 1.7 |
|  | Uncemented | 71 | 231,081 | 77.1 | 67.8 | 73.4 | 64.7 |
| Bearing surface | CoC | 32 | 104,584 | 41.1 | 26.9 | 41.1 | 3.9 |
|  | CoP | 17 | 56,055 | 18 | 16.9 | 21.2 | 4.8 |
|  | MoM | 3 | 8667 | 3.9 | 1.9 | 3.3 | 0.7 |
|  | MoP | 48 | 154,802 | 37 | 54.3 | 34.4 | 90.6 |
|  |  |  |  |  |  |  |  |
| Exchangeable neck | No | 97 | 315,177 | 97 | 97.4 | 97.0 | 98.1 |
|  | Yes | 3 | 8931 | 3.0 | 2.6 | 3.0 | 1.9 |
| Patient characteristics |  |  |  |  |  |  |  |
| Sex | Male | 38 | 122,178 | 100 |  | 42.2 | 23.3 |
|  | Female | 62 | 201,930 |  | 100 | 57.8 | 76.7 |
| Age category (years) | 40-59 | 14 | 46,945 | 21.4 | 10.3 | 17.7 | 4.1 |
|  | 60-74 | 38 | 122,590 | 43.2 | 34.6 | 44.5 | 16.3 |
|  | ≥ 75 | 48 | 154,573 | 35.5 | 55.1 | 37.7 | 79.5 |
| Trauma indication | No | 76 | 246,940 | 85.3 | 70.7 | 100 |  |
|  | Yes | 24 | 77,168 | 14.7 | 29.3 |  | 100 |
| Parkinson disease | No | 96 | 310,950 | 96.4 | 95.6 | 97.5 | 90.9 |
|  | Yes | 4 | 13,158 | 3.6 | 4.4 | 2.5 | 9.1 |
| Diabetes mellitus | No | 88 | 283,633 | 85.1 | 89 | 88.1 | 85.7 |
|  | Yes | 12 | 40,475 | 14.9 | 11 | 11.9 | 14.3 |
| Morbid obesity | No | 92 | 299,430 | 91.2 | 93.1 | 90.6 | 98.1 |
|  | Yes | 8 | 24,678 | 8.8 | 6.9 | 9.4 | 1.9 |
| Treatments |  |  |  |  |  |  |  |
| BZD | No | 50 | 160,819 | 57.9 | 44.6 | 51.3 | 44.3 |
|  | Yes | 50 | 163,289 | 42.1 | 55.4 | 48.7 | 55.7 |
| AH no BZD | No | 86 | 278,574 | 88.1 | 84.7 | 87.1 | 82.2 |
|  | Yes | 14 | 45,534 | 11.9 | 15.3 | 12.9 | 17.8 |
| Antidepressant | No | 77 | 250,690 | 85.8 | 72.2 | 81.8 | 63.1 |
|  | Yes | 23 | 73,418 | 14.2 | 27.8 | 18.2 | 36.9 |
| Antipsychotic | No | 93 | 301,744 | 94.3 | 92.4 | 96.0 | 83.7 |
|  | Yes | 7 | 22,364 | 5.7 | 7.6 | 4.0 | 16.3 |
| Psychostimulant | No | 99 | 320,722 | 99.2 | 98.8 | 99.2 | 98.2 |
|  | Yes | 1 | 3386 | 0.8 | 1.2 | 0.8 | 1.8 |
| Antiosteoporotic | No | 87 | 281,797 | 97.1 | 80.8 | 89.2 | 79.6 |
|  | Yes | 13 | 42,311 | 2.9 | 19.2 | 10.8 | 20.4 |
| Oral corticcosteroïds | No | 74 | 240,910 | 75.9 | 73.4 | 72.2 | 81.3 |
|  | Yes | 26 | 83,198 | 24.1 | 26.6 | 27.8 | 18.7 |
| Hospital characteristics |  |  |  |  |  |  |  |
| Sector | Public | 42 | 136,853 | 39.2 | 44 | 34.0 | 68.6 |
|  | Private | 58 | 187,255 | 60.8 | 56 | 66.0 | 31.4 |
| Number of procedures per month | < 14 | 49 | 158,262 | 49.5 | 48.4 | 49.8 | 45.6 |
|  | 14-38 | 29 | 93,647 | 27.4 | 29.8 | 25.1 | 41.1 |
|  | > 38 | 22 | 72,199 | 23.1 | 21.8 | 25.1 | 13.3 |
| Hospital stay duration (days) | < 6 | 78 | 254,695 | 80.2 | 77.6 | 84.3 | 60.3 |
|  | 6-12 | 5 | 15,952 | 6.2 | 4.1 | 5.7 | 2.3 |
|  | > 12 | 17 | 53,461 | 13.5 | 18.3 | 10.0 | 37.3 |

*Percentages for fixed neck cement types = 101% owing to rounding; CoC = ceramic-on-ceramic; CoP = ceramic-on-polyethylene; MoM = metal-on-metal; MoP = metal-on-polyethylene; BZD = benzodiazepine; AH no BZD = anxiolytic or hypnotic nonbenzodiazepines.
