# Supplementary material for: Exchangeable Femoral Neck (Dual-Modular) THA Prostheses Have Poorer Survivorship Than Other Designs: A Nationwide Cohort of 324,108 Patients
Source: Clin Orthop Relat Res. 2017 Feb 13;475(8):2046–59. doi: 10.1007/s11999-017-5260-6 (PMC5498370; doi:10.1007/s11999-017-5260-6)
Supplement: Supplementary file 2 — Supplementary material 2 (DOC 78 kb) [file 11999_2017_5260_MOESM2_ESM.doc]

**Supplemental Table 2**. Stratified analyses for overall and cause-specific risks of THA revision

|  |  | All-cause revision | | |  |  | Revision for periprosthetic fracture or implant failure | | | |  | Revision for luxation | | | |  | Revision for mechanical complications | | | | |
| --- | --- | --- | --- | --- | --- | --- | --- | --- | --- | --- | --- | --- | --- | --- | --- | --- | --- | --- | --- | --- | --- |
| Variable | Number | Num-ber of  events | Adjus-ted HR^†^ | 95% CI |  | p Value^*^ | Num-ber of  events | Adjus-ted HR^†^ | 95% CI |  | p Value^*^ | Num-ber of  events | Adjus-ted HR^†^ | 95% CI |  | p Value^*^ | Num-ber of  events | Adjus-ted HR^†^ | 95% CI |  | p Value^*^ |
| Overall | 324,108 | 11,968 | 1.26 | 1.14 | 1.38 | < 0.0001 | 1050 | 1.68 | 1.24 | 2.27 | 0.0008 | 2644 | 1.15 | 0.92 | 1.42 | 0.2215 | 7817 | 1.27 | 1.13 | 1.43 | < 0.0001 |
| Stratified on sex |  |  |  |  |  |  |  |  |  |  |  |  |  |  |  |  |  |  |  |  |  |
| Men | 122,178 | 4678 | 1.24 | 1.07 | 1.44 | 0.004 | 336 | 1.58 | 0.93 | 2.67 | 0.089 | 884 | 1.07 | 0.74 | 1.55 | 0.712 | 3110 | 1.28 | 1.08 | 1.53 | 0.006 |
| Women | 201,930 | 7290 | 1.27 | 1.12 | 1.44 | 0.0003 | 714 | 1.72 | 1.19 | 2.05 | 0.0038 | 1760 | 1.19 | 0.91 | 1.56 | 0.2051 | 4707 | 1.26 | 1.08 | 1.48 | 0.0036 |
| Stratified on age group (years) |  |  |  |  |  |  |  |  |  |  |  |  |  |  |  |  |  |  |  |  |  |
| 40-59 | 46,945 | 2203 | 1.27 | 1.05 | 1.54 | 0.014 | 120 | 1.23 | 0.53 | 2.82 | 0.632 | 408 | 1.32 | 0.86 | 2.03 | 0.205 | 1603 | 1.17 | 0.93 | 1.47 | 0.191 |
| 60-74 | 122,590 | 4648 | 1.32 | 1.14 | 1.53 | 0.0002 | 325 | 2.67 | 1.74 | 4.08 | < 0.0001 | 920 | 1.17 | 0.83 | 1.65 | 0.3703 | 3272 | 1.39 | 1.17 | 1.65 | 0.0002 |
| ≥ 75 | 154,573 | 5117 | 1.14 | 0.96 | 1.36 | 0.15 | 605 | 1.14 | 0.68 | 1.91 | 0.63 | 1316 | 0.98 | 0.67 | 1.42 | 0.90 | 2942 | 1.18 | 0.94 | 1.47 | 0.16 |
| Stratified on indication |  |  |  |  |  |  |  |  |  |  |  |  |  |  |  |  |  |  |  |  |  |
| Osteoarthritis | 246,940 | 9043 | 1.25 | 1.13 | 1.39 | < 0.0001 | 648 | 1.86 | 1.31 | 2.65 | 0.0006 | 1738 | 1.13 | 0.88 | 1.45 | 0.3343 | 6340 | 1.25 | 1.10 | 1.41 | 0.0007 |
| Traumatic | 77,168 | 2925 | 1.19 | 0.94 | 1.51 | 0.1456 | 402 | 1.33 | 0.73 | 2.42 | 0.3596 | 906 | 1.13 | 0.72 | 1.76 | 0.5931 | 1477 | 1.32 | 0.97 | 1.80 | 0.0758 |
| Stratified on cementation type |  |  |  |  |  |  |  |  |  |  |  |  |  |  |  |  |  |  |  |  |  |
| Cemented | 34,376 | 1038 | 1.52 | 0.81 | 2.83 | 0.193 | 84 | 5.55 | 1.74 | 17.72 | 0.0038 | 351 | 1.72 | 0.64 | 4.62 | 0.2821 | 583 | 1.45 | 0.60 | 3.50 | 0.4106 |
| Hybrid | 53,611 | 1548 | 1.28 | 0.90 | 1.81 | 0.1716 | 87 | 2.71 | 0.97 | 7.59 | 0.058 | 343 | 0.71 | 0.26 | 1.91 | 0.4923 | 1047 | 1.33 | 0.87 | 2.02 | 0.1835 |
| Reverse hybrid | 5040 | 229 | 1.36 | 0.75 | 2.46 | 0.3145 | 21 | 4.32 | 1.24 | 15.08 | 0.022 | 36 | 1.32 | 0.31 | 5.60 | 0.7059 | 155 | 1.70 | 0.89 | 3.26 | 0.1076 |
| Uncemented | 231,081 | 9153 | 1.25 | 1.13 | 1.39 | < 0.0001 | 858 | 1.45 | 1.03 | 2.04 | 0.0352 | 1914 | 1.17 | 0.93 | 1.47 | 0.1905 | 6032 | 1.26 | 1.11 | 1.43 | 0.0004 |
| Stratified on bearing surface |  |  |  |  |  |  |  |  |  |  |  |  |  |  |  |  |  |  |  |  |  |
| CoC | 104,584 | 4139 | 1.29 | 1.12 | 1.48 | 0.0004 | 273 | 1.32 | 0.76 | 2.28 | 0.3278 | 870 | 1.31 | 0.98 | 1.77 | 0.0734 | 3001 | 1.32 | 1.12 | 1.56 | 0.0009 |
| CoP | 56,055 | 1987 | 1.24 | 0.95 | 1.62 | 0.115 | 171 | 1.96 | 0.95 | 4.04 | 0.0668 | 457 | 1.50 | 0.89 | 2.52 | 0.1304 | 1308 | 1.06 | 0.75 | 1.51 | 0.7362 |
| MoM | 8667 | 433 | 1.25 | 0.88 | 1.79 | 0.2104 | 14 | 0.90 | 0.11 | 7.42 | 0.925 | 81 | 0.13 | 0.02 | 0.92 | 0.0406 | 309 | 1.05 | 0.67 | 1.64 | 0.8363 |
| MoP | 154,802 | 5409 | 1.17 | 0.99 | 1.40 | 0.0712 | 592 | 1.87 | 1.21 | 2.87 | 0.0044 | 1236 | 0.93 | 0.61 | 1.42 | 0.7468 | 3199 | 1.30 | 1.05 | 1.61 | 0.0154 |

^*^Exchangeable neck versus fixed neck THAs; ^†^adjusted hazard ratio of THA revision from multivariate Fine and Gray full Cox model (adjusted for THA characteristics, patient characteristics, treatments, and hospital stay characteristics); HR = hazard ratio; CoC = ceramic-on-ceramic; CoP = ceramic-on-polyethylene; MoM = metal-on-metal; MoP = metal-on-polyethylene.
